# The impact of identifying carotid plaque on addressing cardiovascular risk in psoriatic arthritis

**DOI:** 10.1186/s13075-016-1074-2

**Published:** 2016-08-02

**Authors:** Michael Lucke, William Messner, Esther S. H. Kim, M. Elaine Husni

**Affiliations:** 1 Department of Rheumatology, Allegheny Health Network, Pittsburgh, PA USA; 2 Department of Quantitative Health Sciences, Cleveland Clinic, Cleveland, OH USA; 3 Department of Cardiovascular Medicine, Cleveland Clinic, Cleveland, OH USA; 4 Department of Rheumatic and Immunologic Diseases, Cleveland Clinic, 9500 Euclid Ave, desk A50, Cleveland, OH 44195 USA

**Keywords:** Psoriatic arthritis, Spondyloarthritis, Atherosclerosis, Carotid plaque

## Abstract

**Background:**

Patients with psoriatic arthritis (PsA) are at an increased risk for cardiovascular (CV) disease.  The aim of this study was to identify the frequency of carotid plaque in asymptomatic patients with psoriatic arthritis at baseline and follow-up screening, and to assess for the impact of demonstrating plaque on management of traditional cardiovascular risk factors.

**Methods:**

Eighty-seven PsA patients underwent carotid duplex ultrasound screening. Repeat carotid duplex ultrasound was offered to all patients between 12 and 30 months. Preventive cardiology referrals were generated for all patients through the electronic health record. Traditional cardiovascular risk factors, medication use, and rates of utilization of preventive cardiology services were compared between patients with and without plaque.

**Results:**

Carotid plaque was identified in 34/87 (39 %) of PsA patients. Age and triglyceride levels were predictors of plaque presence. Patients with plaque trended toward higher rates of smoking and diabetes, and higher low-density lipoprotein levels. Only 9/87 (10 %) patients completed at least one visit with preventive cardiology after enrollment despite referral. Low use of statin (21 %) and antiplatelet (27 %) medication was observed. Rates of biologic medication use for PsA were higher (75 %) than studies in similar cohorts of patients with carotid plaque. No association was seen between disease duration or activity and the presence of carotid plaque.

**Conclusion:**

Despite demonstration of high cardiac risk by the presence of carotid plaque, implementation of preventive cardiovascular services and rates of statin and antiplatelet use remained low. Age and triglyceride levels were significant variables in predicting plaque presence. There is no evidence that demonstration of plaque resulted in further evaluation or changes in treatment regimens to address heightened cardiovascular risk.

## Background

Cardiovascular disease risk is elevated in psoriatic arthritis (PsA) compared with the general population and is a major source of morbidity and mortality [1, 2]. The prevalence of traditional risk factors is elevated in both cutaneous psoriasis and PsA compared with the general population [3, 4]. PsA patients suffer from more severe atherosclerosis compared with patients with cutaneous psoriasis [5]. Despite the elevated risk, there remains an unmet need in identifying and addressing CV risk in PsA. There is currently a lack of consensus guidelines in the United States on addressing CV risk in PsA. This study uniquely incorporates both serial carotid ultrasound (US) data in combination with automatic prompting for preventive cardiology referral to a rheumatology clinic population with the goal of assessing the impact of these interventions on CV risk factor modification.

Prior studies have demonstrated suboptimal evaluation and control of modifiable CV risk factors in patients with inflammatory arthritis by both rheumatologists and primary care physicians [6]. Additionally, subclinical atherosclerosis in PsA has been shown to be prevalent even without classic CV risk factors and current risk assessment tools such as the Framingham Risk Score underestimate CV risk in PsA [2, 7, 8], suggesting a role of systemic inflammation in patients with psoriatic skin and joint disease [9, 10]. Prior investigations into the formation of combined cardiology and rheumatology clinics have identified that a high proportion of patients with inflammatory joint pain have indications for medical CV interventions [11], although there were low numbers of PsA patients in this cohort.

Carotid duplex ultrasound (CDU) is a noninvasive imaging technique which can identify the presence of carotid plaque, representing an unequivocal manifestation of atherosclerosis and serving as a surrogate for CV disease. Incorporation of CDU for patients at intermediate risk for CV disease has been used to further enhance CV risk stratification in the general population [12]. Several studies have supported the role of CDU in assessment of CV risk in rheumatoid arthritis, and patients with carotid plaque should be considered as having very high CV risk [13, 14]. In one series of rheumatoid arthritis patients, the presence of bilateral plaque on CDU screening quadrupled the incidence of new acute coronary syndromes independently of any other risk factors compared with patients without carotid plaque [14]. Subclinical atherosclerosis is elevated in patients with PsA compared with controls [1]; screening this population may help identify those patients who are optimal candidates for intensive medical treatment. Prior studies suggest that incorporation of CDU into treatment algorithms can impact progression of plaque and CV outcomes [15]. However, the general applicability of these findings in rheumatology or primary care clinic settings is not clear. Guidelines on the management of patients with identified carotid plaque and PsA have not been formulated.

This study investigates the impact of screening asymptomatic PsA patients for carotid plaque on CV risk factor control. PsA alone is not an indication for antiplatelet or lipid-lowering therapies but should be taken into consideration as part of comprehensive CV risk factor stratification.

## Methods

### Study patients and design

Eighty-seven patients with PsA enrolled in the COMPASS (Cardiometabolic Outcome Measures in Psoriatic ArthritiS Study) database underwent CDU and were screened for the presence of plaque. Patients were ≥18 years old and able to provide consent for enrollment. Patients with a diagnosis of PsA presenting for evaluation in a general rheumatology clinic at a tertiary academic medical center were identified for potential enrollment into the database. PsA was diagnosed after satisfying the criteria of the Classification of Psoriatic Arthritis Study Group with confirmation by a board-certified rheumatologist. Patients remained under the management of their clinical rheumatologist throughout the study.

Referral to preventive cardiology was automatically generated through the EPIC electronic health record as part of best practices management upon study enrollment for all patients. The preventive cardiology clinic at our institution provides complete CV risk assessment and management for both primary and secondary CV disease prevention by a multispecialty team of health care professionals who provide nutritional counseling, exercise instruction, lifestyle and behavioral counseling, smoking cessation programs, medical management of CV risk factors, psychological counseling, and referral to physicians or other health professionals as needed. Extensive assessment of patients’ medical, family, and social history are incorporated with biomarkers and imaging data to risk stratify patients and provide appropriate prevention strategies. Recognizing the heightened CV risk associated with inflammatory arthritides, the preventive cardiology clinic utilizes a primary prevention consensus algorithm for inflammatory arthritis which includes lifestyle counseling for all patients, a goal low-density lipoprotein (LDL) of <100 mg/dl, as well as use of antiplatelet medications in patients with carotid plaque. Rates of completed preventive cardiology consults, defined as one recorded visit with preventive cardiology after enrollment in the COMPASS database, were assessed via electronic record chart review.

### Data collection and assessment

Demographics, traditional CV risk factors, PsA duration, and medication use were recorded at baseline (Table 1) and yearly thereafter using a standardized protocol [16]. CV risk factors addressed included personal history of prior CV events, history of hypertension, history of hyperlipidemia, and history of diabetes. Smoking status was assessed by survey and chart review, and recorded as never, former, or current use, with any ongoing cigarette use defined as a current smoker. Hypertension control was assessed by a cross-sectional recording of last recorded blood pressure during chart review, with 140/90 mmHg used as a cutoff value in accessing control. Body mass index was recorded. Medications assessed included current or prior use of nonbiologic disease-modifying antirheumatic drugs (DMARDS), current or prior biologic DMARDs, nonsteroidal anti-inflammatory drugs (NSAIDS), and steroids. Patients were surveyed on prior dose and duration of prednisone use at enrollment and yearly thereafter. Medication use was confirmed by electronic record chart review at 12–30 months following enrollment. C-reactive protein (CRP), erythrocyte sedimentation rate (ESR), and lipid panels were assessed. When multiple laboratory tests were performed, the most recently performed test after entry into the cohort was used. Additional laboratory tests ordered by preventive cardiology clinics were recorded via electronic record chart review. A 68/66 tender/swollen joint count was performed by a physician, nurse practitioner, or physician assistant with training in performing joint counts.

**Table 1:** Comparisons of variables between patients with and without plaque

		No plaque		Plaque		
Factor	Total *N* assessed	*N*	Statistics	*N*	Statistics	*p* value^a^
Age^b^	87	53	49.58 ± 10.56	34	58 ± 8.58	<0.001^T^
Male sex^c^	87	26	49.06	15	44.12	0.82^C^
History of hypertension^c^	86	17	32.69	23	67.65	0.003^C^
BP < 140/90 mmHg^c^	87	42	79.25	20	58.82	0.07^C^
Smoking status^c^	87					0.052^C^
No	44	29	54.72	15	44.12	
Former	26	18	33.96	8	23.53	
Current	17	6	11.32	11	32.35	
BMI^b^	79	47	30.87 ± 8.54	32	30.34 ± 6.16	0.75^T^
Diabetes^c^	87	7	13.21	9	26.47	0.20^C^
Total cholesterol^b^	75	44	184.5 ± 29.31	31	202.48 ± 45.16	0.057^T^
Triglyceride^d^	74	43	95 (70, 125)	31	144 (87.5, 177.5)	0.014^W^
HDL^b^	74	43	55.12 ± 15.49	31	56.16 ± 21.55	0.82^T^
LDL^b^	74	43	104.07 ± 26.35	31	118.74 ± 41.94	0.092^T^
Antiplatelet use^c^	87	14	26.42	10	29.41	0.95^C^
Statin use^c^	87	9	16.98	10	29.41	0.27^C^
Biologic use^c^	87					0.03^F^
No	17	14	26.42	3	8.82	
Former	5	1	1.89	4	11.76	
Current	65	38	71.7	27	79.41	
Traditional DMARD use^c^	87					0.75^F^
No	11	7	13.21	4	11.76	
Former	34	19	35.85	15	44.12	
Current	42	27	50.94	15	44.12	
History of CVD^c^	87	2	3.77	5	14.71	0.11^F^
ESR^d^	69	37	8 (4, 10)	32	8 (5.25, 12.25)	0.53^W^
CRP^d^	84	52	0.3 (0.1, 0.6)	32	0.3 (0.2, 1.22)	0.11^W^
PsO duration^b^	85	52	21.02 ± 14.79	33	22.58 ± 19.14	0.69^T^
PsA duration^b^	84	51	10.76 ± 8.32	33	12.76 ± 10.7	0.37^T^
Prednisone use > 1 month^c^	86	26	49.06	11	33.33	0.23^C^
Current NSAID use^c^	87	27	50.94	22	64.71	0.30^C^
Baseline SJC^d^	81	49	1 (0, 5)	32	0 (0, 4.5)	0.88^W^
Baseline TJC^d^	81	49	5 (0, 14)	32	5 (0.75, 16.25)	0.93^W^

^a^ *C* Pearson’s chi-squared test with Yates’ continuity correction, *F* Fisher’s exact test for count data, *T* Welch Two Sample T-test, *W* Wilcoxon rank sum test with continuity correction

^b^Mean ± SD

^c^Percentage

^d^Median (25th percentile, 75th percentile)

*BP* blood pressure, *CRP* C-reactive protein, *CVD* cardiovascular disease, *DMARD* disease-modifying antirheumatic drug, *ESR* erythrocyte sedimentation rate, *HDL* high-density lipoprotein, *LDL* low-density lipoprotein, *NSAID* nonsteroidal anti-inflammatory drug, *PsA* psoriatic arthritis, *SJC* swollen joint count, *TJC* tender joint count *PsO* psoriasis

The multivariate logistic regression model including age and triglycerides was performed (Table 2). When controlling for age and triglyceride levels in a multivariate setting, hypertension is not significant (*p* = 0.13). However, age and triglycerides remain significant. The probability of observing carotid plaque increases with higher levels of both these variables.

**Table 2:** Multivariate logistic regression model for identification of carotid plaque

Term	Coefficient	Standard error	*p* value	Odds ratio	95 % CI on odds ratio
Intercept	6.59	1.69	<0.001	–	–
Age	0.0936	0.0281	<0.001	1.098	1.043–1.166
Triglycerides	0.0087	0.00401	0.03	1.009	1.001–1.018

### Ultrasound imaging

**Fig. 1:**
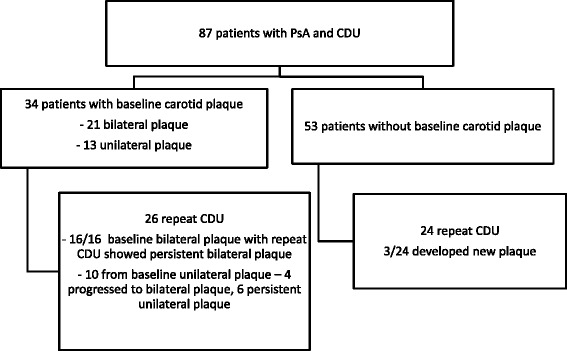
Flow diagram. *CDU* carotid duplex ultrasound, *PsA* psoriatic arthritis

A standardized protocol was performed by registered vascular sonographers who had been specifically trained in CDU imaging using the Philips iU22 US system with an 8–14 MHz linear transducer. A circumferential plaque screen including assessment of internal carotid, external carotid, and common carotid arteries was performed using both transverse and longitudinal views of the bilateral carotid arteries with plaque in any vessel recorded as a positive result. Carotid plaque was defined using the Mannheim consensus criteria [17]. Repeat CDU was offered to all 87 patients through telephone calls and was performed between 12 and 30 months in 50 patients (Fig. 1). Results of US imaging, reported as normal or abnormal, were sent to patients as per IRB protocol. All patients were also given an opportunity to allow release of results to their primary care physicians and rheumatologists.

### Statistical analyses

Continuous variables were described using means and standard deviations or medians and interquartile ranges. Categorical variables were described using counts and percentages. Comparisons of continuous variables between groups were made using either Welch’s two-sample *t* test or Wilcoxon’s rank-sum test. Comparisons of categorical variables between groups were made using Pearson’s chi-squared test or Fisher’s exact test. Multivariate logistic regression modeling was used to measure relationships between patient CV risk factors and the presence of carotid plaque (Table 2). Where relevant in the multivariate modeling, missing values were handled by median imputation. All analyses were done using R software (version 3.0.2; Vienna, Austria). All testing was two-sided and considered significant at the 0.05 level.

## Results

Carotid plaque was identified in 34/87 (39 %) of patients with PsA. Older age (*p* < 0.001), history of hypertension (*p* < 0.003), and triglyceride levels (*p* < 0.014) were statistically significant variables associated with the presence of carotid plaque (Table 1). A higher percentage of patients with plaque were current smokers (32 % versus 11 %). Patients with carotid plaque trended towards higher LDL levels compared with those without plaque (124 versus 102 mg/dl). Length of cutaneous psoriasis and PsA, as well as ESR and CRP, were not statistically significantly different between groups. Swollen joint count (SJC) and tender joint count (TJC) were comparable between groups, with a median of five tender joints in both groups and of zero and one swollen joint in the no plaque and plaque groups respectively.

Because the three significant CV risk factors (age, hypertension, and triglyceride levels) were intercorrelated, multivariate logistic regression modeling was implemented to limit possible confounding. Controlling for age and triglyceride levels, hypertension was not significant (*p* = 0.13). The multivariate logistic regression model including age and triglyceride as predictors demonstrates increased probability of observing carotid plaque with higher levels of these two variables.

Twenty-one patients had bilateral plaque present at baseline screening. Of the 16/21 patients with bilateral plaque and repeat carotid CDU screening, all demonstrated persistent plaque. Thirteen patients had unilateral plaque at baseline. Of the 10 patients with unilateral plaque who had a repeat CDU, 4/10 (40 %) showed progression from unilateral to bilateral plaque. A total 3/24 (12.5 %) of patients without plaque at baseline who had repeat CDU performed showed new plaque presence.

Only nine patients completed preventive cardiology appointments after enrollment in the COMPASS database. There was no evidence of a difference in the rates of utilization of preventive cardiology between patients with PsA without carotid plaque and those with plaque (*p* = 0.73). Four patients (11.7 %) with baseline plaque completed evaluations compared with five patients (9.4 %) without baseline plaque. Note that the 95 % confidence interval for the odds ratio between the groups is quite wide (0.234, 6.460), indicating that while there is no evidence of a difference, a meaningful difference cannot be ruled out. Routine interventions for these patients included a comprehensive assessment of diet, weight, and exercise. In addition to standard lipid profiles, a “preventive profile” consisting of lipoprotein (a), lipoprotein-associated phospholipase A2, fibrinogen, myeloperoxidase, NT-pro BNP, high-sensitivity CRP, and urine albumin:creatnine ratio was checked in 13/16 patients who saw preventive cardiology during or prior to study enrollment and were assessed to have elevated CV risk. Fourteen other patients were seen by a cardiologist for other reasons including hypertension, syncope, arrhythmias, and valvular heart disease.

High percentages (75 %) of our cohort were currently being treated with a biologic DMARD, the majority of which were on anti-TNF-α medications except for two patients on ustekinumab. Rates of biologic medication use in our cohort are significantly higher than in other studies on subclinical atherosclerosis in PsA; prior rates of biologic DMARD use range from 1% [7] to 51 % [9]. More patients with plaque were treated with biologic medications while more patients without plaque were taking a traditional DMARD. Methotrexate was the medication of choice in the vast majority of patients on a DMARD.

Only four patients with plaque had a prior history of CV disease, two of these patients were on maximum dose statins. No other patient in the study population was treated with maximum dose statins. Two patients without plaque had a history of CV disease (one cerebrovascular accident, one non-ST elevated myocardial infarction).

## Discussion

Despite noninvasive screening demonstrating high rates of carotid plaque, few PsA patients completed consultations with the preventive cardiology service. There is no evidence that patients with carotid plaque utilized preventive cardiology visits more than those patients without plaque. This study incorporates multiple strategies to address CV risk in PsA through incorporation of both CDU and automatic referrals to preventive cardiology. Despite this, a minority of patients with and without carotid plaque met the stated LDL goals and few were on antiplatelet therapy.

A goal of electronic health records is to enhance meaningful use and allow identification of high-risk groups. In this study, incorporation of automatic “best practice” electronic referrals was not an effective strategy to increase rates of specialty CV screening, even with a preventive cardiology clinic available in the same institution. This additional optional electronic alert may have been perceived as too time consuming or not of high value in patient care by physicians, and may not be of high utility to improve outcomes as a general treatment strategy.

There was a high prevalence of traditional cardiac risk factors in our cohort; 8 % had a history of prior CV disease, 18 % were diabetic, 46 % had a history of hypertension, and 49 % had a history of smoking. Rates of referral completion were not changed by demonstrating high CV risk through detection of carotid plaque. Lack of appreciation of CV risk by patients, primary providers, and specialists may all contribute to the low referral rate. In addition to traditional risk factors, demonstration of carotid plaque can improve risk stratification but will only be impactful if incorporated into treatment plans by primary care physicians, rheumatologists, and/or cardiologists. The absence of guidelines on the use of this screening measure may have contributed to the lack of interventions in our group.

Identification of patients with PsA who may benefit most from the addition of CDU to traditional risk factor assessment is critical for meaningful implementation of this additional screening test. Early establishment of the level of CV risk has the potential to improve CV outcomes, but presently it is unclear how to best screen these patients with elevated baseline risk. Our results indicate that age and high triglyceride levels are associated with the presence of plaque, and smoking was more common in patients with plaque. As expected, the incidence of plaque increased with age. The 25th percentile for age in patients with plaque in our cohort was 51 with a median of 55. CDU may be a helpful risk stratification tool to assist in risk-prevention strategies in patients with PsA, particularly in patients over 50 years old requiring biologic use.

Prevalence of plaque in our PsA population is higher than in some PsA cohorts [7, 18], lower than other series [9], and similar to a rheumatoid arthritis cohort [19]. Rates of plaque in inflammatory arthritis have been shown to be increased compared with age-matched controls in the general population [20]. The high rates of plaque in PsA support a role for CDU in select PsA patients to identify high CV risk patients who lack traditional risk factors or overt CV disease. While an interval of 12–24 months may be too short to detect progression of atherosclerosis in some patients, new formation of plaque after 1–2 years of follow up in three patients suggests a potential role for serial monitoring of high-risk patients with CDU as well as early medical intervention. The 40 % (4/10 patients) rate of progression from unilateral plaque to bilateral plaque over 1–2 years suggests a role for medical interventions at the time of discovery of plaque but will require confirmation in a larger sample size. Lipid levels did not always correlate with plaque progression, as these three patients’ LDL ranged from 73 to 187 mg/dl.

ESR and CRP levels have not been shown to be reliable measures of active disease in PsA and were not associated with plaque formation in our study, which did not use a high-sensitivity CRP assay and was limited by the use of a cross-sectional measurement. No significant difference in the prevalence of plaque and disease duration was found, which has also been noted in a recent publication [9]. Patients in our cohort had a high variability in the duration of disease. It is possible that anti-inflammatory treatments mitigated the effect of chronic inflammation associated with longer disease activity. Rates of biologic medication use in our cohort are significantly higher than other studies on subclinical atherosclerosis in PsA; prior rates of biologic DMARD use range from 1 % [7] to 51 % [9].

Although conflicting results have been reported, some studies have suggested that TNF-α antagonists may have a beneficial role in preventing progression of subclinical atherosclerosis [21]. A prior series of PsA patients showed a lower prevalence of carotid plaques in patients on TNF-α blockers compared with nonbiologic DMARDS [22]. In our cohort, there was a higher prevalence of biologic use in the carotid plaque group, which may be a reflection of higher disease activity and a tertiary center patient population.

The low sample size in this exploratory study of combining screening measures is a limitation. Another limitation of our cross-sectional study is an inability to access cardiac interventions performed outside our institution. As a retrospective chart review, some data including BMI and cholesterol panels were unavailable for analysis. In patients without plaque, 88 % had BMI data and 83 % had a cholesterol panel, versus 94 % and 91 % respectively of patients with plaque. The extent of cutaneous psoriasis was not measured between the groups.

The time frame of follow up may be too short to evaluate for changes in plaque formation. However, even over a short interval several patients demonstrated new plaque, suggesting an accelerated risk of CV events. The low total number of patients with new plaque limits the generalizability of this finding as well as further analysis of risks for progression. Likewise, there is limited power to analyze for efficacy of the preventive cardiology program in modifying CV risk factors between those who completed and did not complete an appointment. Low numbers of clinical CV events were seen, probably due to short duration of follow up. While the presence of plaque is a strong indicator of heightened CV risk, other variables such as plaque characteristics and change in plaque size were not assessed. Dose and duration of prednisone use and NSAID use were reliant upon patient survey and recollection. Dependence on patient survey responses were reduced by simultaneous medical record review to confirm medications and documented CV risk factors. The cross-sectional nature of the study did not allow for an assessment of the inflammatory burden of PsA over time.

The low rates of preventive cardiology clinic attendance despite automatic referral merits further investigation. It is possible that debate over the level of CV risk in PsA limited physician responses to electronic referral recommendations, which could be declined easily by the physician. Physicians may have been satisfied with control of the disease and did not perceive these patients to be at elevated risk. Lack of familiarity with CDU screening and the risk represented by carotid plaque may have also led to an under-appreciation of CV risk. While the results of the US scan were provided to the patient’s primary rheumatologist and the patient, an interpretation of the findings regarding level of CV risk was not provided. Because the US examinations were conducted outside of a standard clinic visit, the results may not have been incorporated into the physician’s assessment at the time of follow-up rheumatology visits. The distance required for return visits to a tertiary care center also likely impacted the percentage of patients completing preventive cardiology referrals. Physicians and patients, especially those with prior cardiology clinic visits, may not be aware of the multispecialty approach of the preventive cardiology clinic. It is possible that patients sought a cardiology opinion at a local facility; the study design did not collect additional information about interval visits with a local cardiologist.

Further evaluation into the barriers of addressing CV risk in PsA patients is required. CV risk stratification and management in PsA may be improved by incorporation of carotid CDU to assess for carotid plaque. The presence of a preventive cardiology program is not widely available, but even with the presence of this program at our institution it was underutilized by a high-risk population. Combined cardiology–rheumatology clinics have shown promise, with one study reporting successful treatment to lipid targets in 90 % of inflammatory joint pain patients referred to the combined clinic [11]. Translating identification of elevated CV risk in PsA patients into preventive interventions to reduce risk remains a challenge. With increasing evidence of this risk identified over recent years, development of further guidelines to address modifiable risk factors in this patient group is needed.

## Conclusion

Interventions to aggressively assess and modify CV risk in PsA remain an unmet aspect of disease management despite the high morbidity and mortality of CV disease in this population. Rates of carotid plaque are high in PsA and patients with carotid plaque should be considered as having very high CV risk. In our cohort, patients aged over 50 with elevated triglyceride levels are at higher risk for development of carotid plaque. Despite demonstration of high cardiac risk by carotid plaque, implementation of preventive CV strategies in PsA patients remained poor. This study emphasizes the need for further consensus in establishing protocols for CV risk assessment in PsA.

## Abbreviations

CDU, carotid duplex ultrasound; COMPASS, Cardiometabolic Outcome Measures in Psoriatic ArthritiS Study; CRP, C-reactive protein; CV, cardiovascular; DMARD, disease-modifying antirheumatic drug; ESR, erythrocyte sedimentation rate; LDL, low-density lipoprotein; NSAID, nonsteroidal anti-inflammatory drug; PsA, psoriatic arthritis; SJC, swollen joint count; TJC, tender joint count, US, ultrasound
